# The Role of Inhibitory Control Mechanisms in the Regulation of Sexual Behavior

**DOI:** 10.1007/s10508-018-1283-7

**Published:** 2019-01-22

**Authors:** Geraldine Rodriguez-Nieto, Franziska Emmerling, Marieke Dewitte, Alexander T. Sack, Teresa Schuhmann

**Affiliations:** 1 Department of Cognitive Neuroscience, Faculty of Psychology and Neuroscience, Maastricht University, Oxfordlaan 55, 6229 EV Maastricht, The Netherlands; 2 Maastricht Brain Imaging Center, Maastricht University, Maastricht, The Netherlands; 3 Department of Experimental Psychology, University of Oxford, Oxford, UK; 4 Department of Clinical Psychological Science, Faculty of Psychology and Neuroscience, Maastricht University, Maastricht, The Netherlands

**Keywords:** Sexual inhibition, General inhibition, Go/No-go task, Approach–Avoidance task, Priming inhibition

## Abstract

Sexual behavior is the open manifestation of a complex interplay between psychophysiological mechanisms that either facilitate or inhibit sexual thoughts, desires, and associated behaviors. Whereas sexual excitation has been widely studied, less is known about the impact of inhibitory control mechanisms that enable individuals to refrain from sexual cognition and behavior. The present study examined: (1) the relationship between general and sexual inhibitory mechanisms (as measured through self-reports and computer-based tasks), (2) the relation between sexual inhibitory processes at cognitive and motor-motivational levels and with sexual inhibition as an individual trait, and (3) the predictive value of these parameters on sexual thoughts (cognition) and behavior. We demonstrate that general inhibitory control (i.e., the ability to suppress any preponderant response) and the specific inhibition of sexual responses represent distinct processes that require at least partly different control mechanisms. Similarly, the ability to inhibit sexual visual input and the ability to suppress sexually driven responses seem to be two independent processes. The different inhibitory processes distinctively predicted the frequency of sexual thoughts and sexual behavior. We propose that these different inhibitory mechanisms are at play during different phases of sexual regulation (before and after the generation and unfolding of sexual arousal) and that a specific deficit in one of these processes may underlie the distinctive symptomatology and comorbidity of sexual disorders.

## Introduction

Human sexuality is an essential part of our existence and comprises a broad range of aspects, including cognitive, emotional, behavioral, and physiological processes. These processes are modulated by a complex interplay between inhibitory (restrain, cancel, suppress) and excitatory (initiate, execute, promote) mechanisms, shaping the manifestation of sexual thoughts (cognition), desire (motivational state), and behavior. The successful inhibition of sexual expressions is a natural and adaptive reaction to contextual factors (Bancroft, Loftus, & Long, 2003) and enables the regulation of sexual behavior, thereby preventing undesired consequences for the individual and for society.

In spite of their relevance, sexual inhibitory mechanisms have been scarcely studied. When trying to understand sexual inhibition, it is important to specify its defining characteristics and differentiate it from other types of inhibition. A central question is whether the ability to regulate sexual cognition and behavior overlaps with an individual’s general ability of behavioral control or whether it recruits inhibitory mechanisms specific to sexual behavior. On the one hand, there is evidence showing that both types of inhibition are interrelated. For example, efficient response inhibition of sexual stimuli was found to be inversely related to general impulsivity (Macapagal, Janssen, Fridberg, Finn, & Heiman, 2011). Likewise, high self-reported impulsivity was found to be related to a higher frequency of risky sexual behaviors (Curry et al., 2018; McCoul & Haslam, 2001), sexual compulsivity/hypersexuality (Miner, Raymond, Mueller, Lloyd, & Lim, 2009; Miner et al., 2016), low sexual restraint, and inclination toward infidelity (Gailliot & Baumeister, 2007). Additionally, the comorbidity of sexual disorders with other inhibition-related disorders is not uncommon (e.g., ADHD, substance abuse; Bancroft & Vukadinovic, 2004). Further evidence comes from frontal brain damage patients, who sometimes exhibit inappropriate sexual behavior in the context of general disinhibition (Baird, Wilson, Bladin, Saling, & Reutens, 2007).

There is, however, also evidence pointing to the specificity of sexual inhibition, independently of general inhibition. For example, damage to non-frontal brain areas have led to specific forms of sexual disinhibition, such as compulsive masturbation, intrusive sexual thoughts, or sexual addiction in the absence of general inhibition deficits (Baird et al., 2007).

In addition, different sexual disorders are characterized by specific forms of sexual disinhibition. For example, while paraphilias are often characterized by obsessive sexual thoughts, pornography addiction leads to a compulsive urge to watch pornography, and some forms of sexual addiction can lead to a promiscuous life, endangering health and personal and social relationships (Bancroft & Vukadinovic, 2004; Love, Laier, Brand, Hatch, & Hajela, 2015; Vella-Zarb, Cohen, McCabe, & Rowa, 2016). This suggests the involvement of various and specific inhibitory mechanisms in the regulation of sexual behavior.

To address the possibility of different sexual inhibitory processes, we refer to the process model of emotion regulation (Gross, 2002). Given that sexual arousal can be regarded as an emotion (Everaerd, Both, & Laan, 2006), this framework has a clear heuristic value for understanding sexual responding. According to this model, individuals can modulate their emotions either before they unfold via cognitive pathways, such as attention deployment or reappraisal, or after the emotions have unfolded via the suppression of the behavioral response. Accordingly, when an individual is aware of the risk of getting sexually aroused in inappropriate circumstances, he or she might redirect and focus the attention toward non-sexual stimuli or change the appraisal of the situation; but when the sexual arousal has initiated, it may be better suited to use avoidant actions or suppression mechanisms. We propose that these different regulatory processes, occurring at different phases in the regulation of sexual behavior, imply the existence of different inhibitory mechanisms, which in turn differentially overlap with certain aspects of one’s general ability of inhibitory control.

However, in order to understand the regulation of the sexual response, not only the inhibitory mechanisms are relevant. According to the dual control model of male sexual response, it is the balancing between sexual inhibitory and sexual excitatory mechanisms that allows an adaptive sexual response consonant with the circumstances (Bancroft & Janssen, 2000). Whereas sexual excitation refers to the propensity to get sexually aroused or get an erectile response, sexual inhibition refers to processes that diminish that response.

Individuals vary in the degree to which they are sexually aroused and inhibited and these traits have been measured by the sexual excitation and inhibition scales (Janssen, Vorst, Finn, & Bancroft, 2002). Regarding sexual inhibition, two factors can be distinguished: SIS1—inhibition due to threat of performance failure and SIS2—inhibition due to the threat of the consequences of the performance (Bancroft & Janssen, 2000). These factors measure psychophysiological traits, but far less is known about sexual inhibitory mechanisms measured as cognitive processes. Studying the basic processing of sexual inhibition is fundamental to understand the underlying mechanistic failure that leads to undesired consequences and sexual disorders.

### Sexual Inhibition and General Inhibition

In this study, we primarily aimed at testing whether general inhibition relates to sexual inhibition measured as traits and as processes. To study general and sexual inhibition traits, we selected the Brief Self-Control Scale (BSCS; Tangney, Baumeister, & Boone, 2004), which measures a general ability to control own behavior, and the second factor of the Sexual Inhibition Scale (SIS2; Janssen et al., 2002). We did not consider the first factor (SIS1) because it likely comprises a peripheral component (reflects the amount of effort needed to maintain an erection) and thus may reflect more a physiological-dependent process (Bancroft, Graham, Janssen, & Sanders, 2009). In contrast, the second factor (SIS2) constitutes an adaptive mechanism to prevent harmful consequences derived from the sexual performance (e.g., loss of arousal under the risk of getting a sexually transmitted disease).

To study general inhibition as a process, we selected the classic Go/No-go paradigm, in which individuals are required to restrain from responding to an infrequent stimulus. To assess sexual inhibition, we used the Approach–Avoidance (AA) task to measure the control over a motivationally driven motor response, thus comprising a peripheral inhibitory component. We formulated the following propositions:

#### **Hypothesis 1**

Sexual motor inhibition (AA) relates to general motor response inhibition (measured by the Go/No-go task) as both processes require withholding a dominant motor response.

#### **Hypothesis 2**

The second factor of Sexual Inhibition (SIS2) trait relates to general self-control (BSCS) as both traits comprise the ability to withhold actions that lead to pleasurable consequences by means of higher-order processing such as preventing negative consequences or pursuing long-term goals.

### Sexual Motor Inhibition, Sexual Cognitive Inhibition, and Sexual Inhibition as Trait

We further aimed to test whether different sexual inhibition processes relate to each other and to sexual inhibition as trait, or whether they comprise distinct constructs. As we proposed that sexual inhibition does not compromise a unitary process and that different mechanisms withhold the inhibition at different phases of the cascade of sexual arousal responding, we used two distinct paradigms to assess sexual inhibition. In addition to the AA task, which measures sexual motor inhibition, we selected the Negative Affective Priming (NAP) task. This task allowed us to study sexual inhibition at a central cognitive level, as subjects are required to ignore an affective distractor while attending a non-affective target; in a post-trial, the subjects are required to respond to a stimulus with the same content type than the one that was previous inhibited, which causes a response delay compared to non-priming trials. We expected:

#### **Hypothesis 3**

The two sexual inhibitory processes are not related, as one comprises cognitive processing (NAP) and the second (AA) comprises a rather “hot” component, as individuals are required to either approach or avoid sexual stimuli.

#### **Hypothesis 4**

The second factor of Sexual Inhibition relates to sexual motor inhibition as process (AA) since both constructs imply the control over sexually rewarding actions and thus comprise a strong motivational and peripheral component.

### Regulatory Mechanisms of Sexual Thoughts and Sexual Behavior

Finally, since we hypothesized that different inhibitory mechanisms play a distinct role at different levels of the sexual arousal unfolding, and because sexual responses and related behaviors result from the balance between excitatory and inhibitory mechanisms, we tested whether different general and sexual inhibition traits and processes along with sexual excitation (measured by the Sexual Excitation Scale; Janssen et al., 2002) predicted the frequency of typical sexual behaviors.

For this purpose, we selected sexual manifestations aimed to distinguish a cognitive and a behavioral component. Moreover, within the behavioral level, we distinguished between solitary and dyadic manifestations as dyadic sex involves social cognition and therefore may involve different inhibitory mechanisms. Specifically, we selected the frequency of sexual thoughts, for being a sexual process which does not manifest itself behaviorally; pornography watching, for being a private motivationally driven behavior; masturbation, for being a private behavior with a peripheral physiological output; and intercourse, for being a sexual behavior with a social component. We considered the frequency of those behaviors an indicator of the balance of the regulatory mechanisms in healthy conditions, as very high or very low frequencies could lead to undesired consequences or dissatisfaction. In particular, we predicted that:

#### **Hypothesis 5**

Sexual cognitive inhibition (NAP) predicts the frequency of sexual thoughts as sexual cognitive inhibition reveals the control over central processes through attention.

#### **Hypothesis 6**

The sexual motor inhibition (AA) and general inhibition processes (Go/No-go) relate directly to sexual behavior since they reflect the motor control over preponderant responses.

#### **Hypothesis 7**

Sexual inhibition (second factor) and general inhibition (self-control) as traits have predictive value regarding sexual behavior, as both constructs measure the ability to control pleasurable actions at the foresight of long-term consequences.

#### **Hypothesis 8**

Sexual excitability is a significant predictor of sexual thoughts and sexual behavior.

As women and men respond differentially during sexual cognitive and inhibitory processes (Dewitte, 2016; Sjoberg & Cole, 2018) and because women are less vulnerable to sexual inhibition impairments such as in hypersexuality (Kuzma & Black, 2008), we included only men in our sample to provide a first test of the role of inhibition in sexual responding.

## Method

### Participants

A total of 52 self-declared heterosexual male participants (18–35 years old; *M* age = 24.46; SD = 4.32) were recruited through advertising in Maastricht University halls and in a Facebook group created to recruit participants for research at Maastricht University. All of the participants were financially reimbursed for their participation with 10 euros in vouchers. The study was approved by the local ethical committee. A total of 90.3% of participants were Bachelor or Master students; from the rest, three participants had completed university and two had completed high school. Approximately half of the participants were single (53.8%); the others had been in a relationship for an average period of 2.7 years (SD = 2.85). Single and coupled men did not differ in age nor in individual traits (sexual excitability, sexual inhibition, and self-control) measured by self-reports.

### Procedure and Measures

After giving their informed consent, participants performed three computer-based tasks: two sexual (Negative Affective Priming task and Approach–Avoidance task) and one non-sexual (Go/No-go task). The order in which the two sexual tasks were presented was counterbalanced. The Go/No-go task was always presented between the sexual tasks to prevent habituation to sexual stimuli. The questionnaires were completed at the end of the session. To increase the confidence of participants on the anonymity of their answers and increase their comfort, their identities were registered as number codes and they were left alone in the room while answering.

#### Negative Affective Priming Task (NAP)

Our version of the task (adapted from Dewitte, 2016) consisted of three blocks containing 32 trial sequences each. There were four types of trial sequences which were presented randomly in equal proportion throughout each block. Each trial sequence consisted of a prime trial and a probe trial. During each, two pictures were simultaneously presented, one surrounded by a black frame and the other by a gray frame. Images were presented above each other. Participants had to attend only to the black frame picture (target) while ignoring the one with the gray frame (distractor) and to indicate whether the target displayed sexual or non-sexual content by button press.

**Fig. 1:**
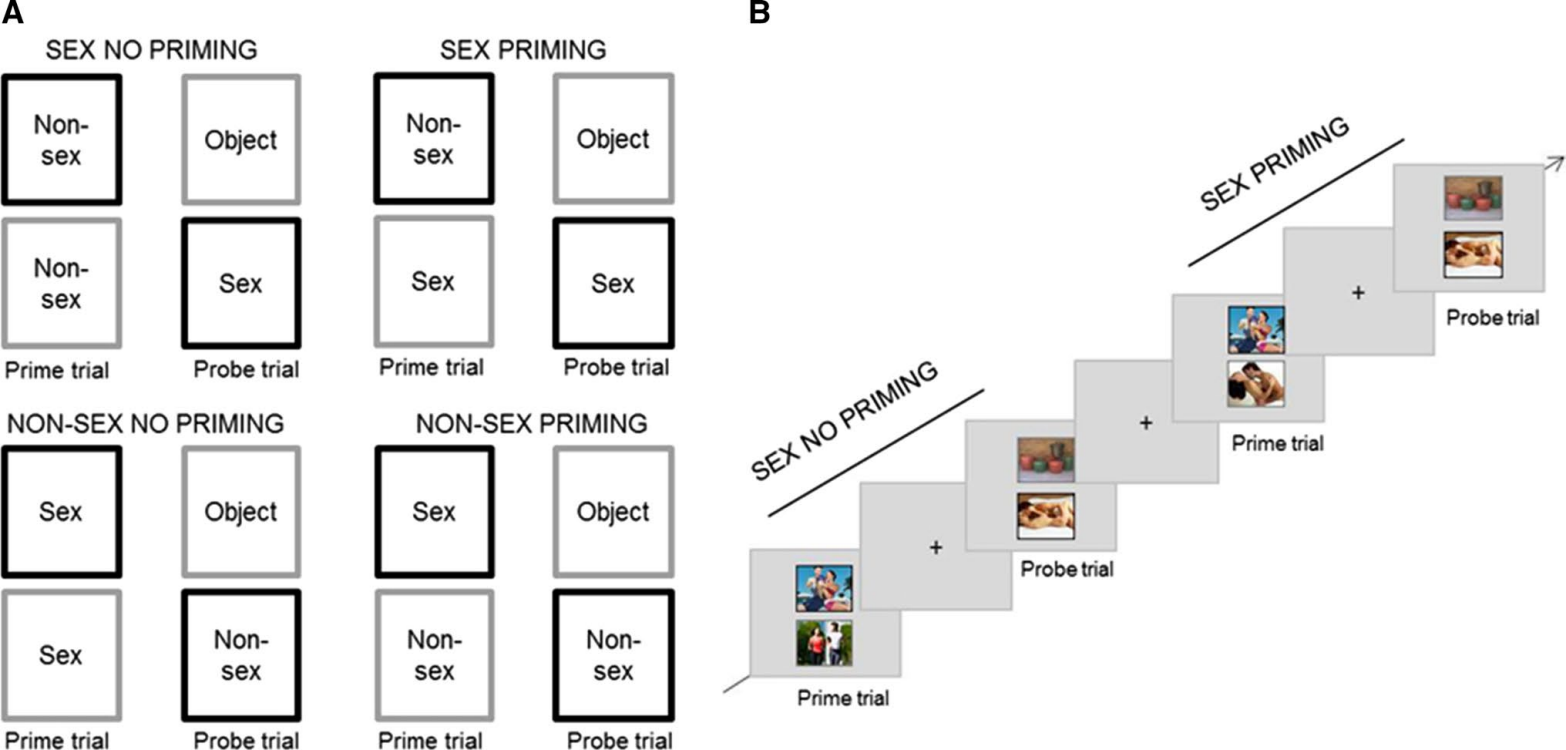
Negative Affective Priming task (NAP). **a** Four conditions: In the two priming conditions (sex and non-sex), the content of the distractor in the prime trial matched the content of the target in the probe trial. In the no priming conditions, the content of the distractor in the prime trial was different from the content of the target in the probe trial. **b** Timeline: example of a Sex Priming and a Sex No Priming trial. The stimuli shown are dummy photographs only used for illustrative purposes

During the experimental trial sequences (Priming), the content of the distractor picture in the prime trial matched the content type of the target in the probe trial, whereas in the control trial sequences (No Priming) the content of these two pictures differed. The target in the probe trial could be sexual or non-sexual. This way, there were four different conditions: Sex Priming (sexual distractor in the prime trial with a sexual target in the probe trial), Sex No Priming (non-sexual distractor in the prime trial with a sexual target in the probe trial), Non-Sex Priming (non-sexual distractor in the prime trial with a non-sexual target in the probe trial), and Non-Sex No Priming (sexual distractor in the prime trial with a non-sexual target in the probe trial) (Fig. 1a). The prime and probe trials were presented for 1750 ms each, separated by a fixation cross presented for 1750 ms. The interval between the trial sequences was filled with the same fixation cross for 1750 ms and was not jittered so that the participants did not identify that the prime and probe trials were part of a predefined trial sequence (Fig. 1b). The sexual stimuli were pictures (320 × 260 pixels) displaying sexual intercourse or oral sex between one man and one woman. The non-sexual stimuli were color pictures of one man and one woman exercising together. The neutral stimuli (distractors in the probe trials) were pictures of neutral objects (e.g., light switch). Pictures were displayed on light gray background, and the picture frames were three pixels thick. Most of the images (85%) were drawn from validated data sets that were evaluated on the basis of valence and arousability (Dewitte, 2016; Rupp & Wallen, 2007). As there were not enough available images from the previous data sets, the remaining images were selected from the Internet and approved by three judges considering that the content was similar to those of the rated photographs, mainly regarding body postures, positions, and dimensions.

As the inhibitory effect is implied from the response delay in priming conditions, we calculated a sexual priming index by subtracting the reaction times in Sex No Priming trials to those of Sex Priming trials. Because we were specifically interested in the sexual priming effect, we subtracted a non-sexual priming index to the sexual priming index ([Sex Priming–Sex No Priming]–[Non-Sex Priming–Non-Sex No Priming]) to exclude the non-sexual inhibitory component. This index was the main score used for this task. A higher index indicated a stronger sexual priming effect and thus a stronger sexual inhibition.

#### Approach–Avoidance Task

This task consisted of four blocks with 48 stimuli each, half of them of sexual content and the rest with non-sexual content (adapted from Dewitte, 2016). Participants were told that in some blocks they would have to approach the sexual stimuli and avoid the non-sexual ones, whereas in other blocks they would have to do the opposite. To approach a stimulus, they were instructed to move the joystick toward them, mentioning that this implied the notion to bring the stimulus closer. With doing so, the image size increased. To avoid the stimulus, they had to push the joystick away from them, which implied the notion of pushing the stimulus away, and decreased the image size. They performed 20 practice trials, using plants and animals as stimuli, so that they got acquainted with associating the movement of the joystick with the respective resizing of the image.

**Fig. 2:**
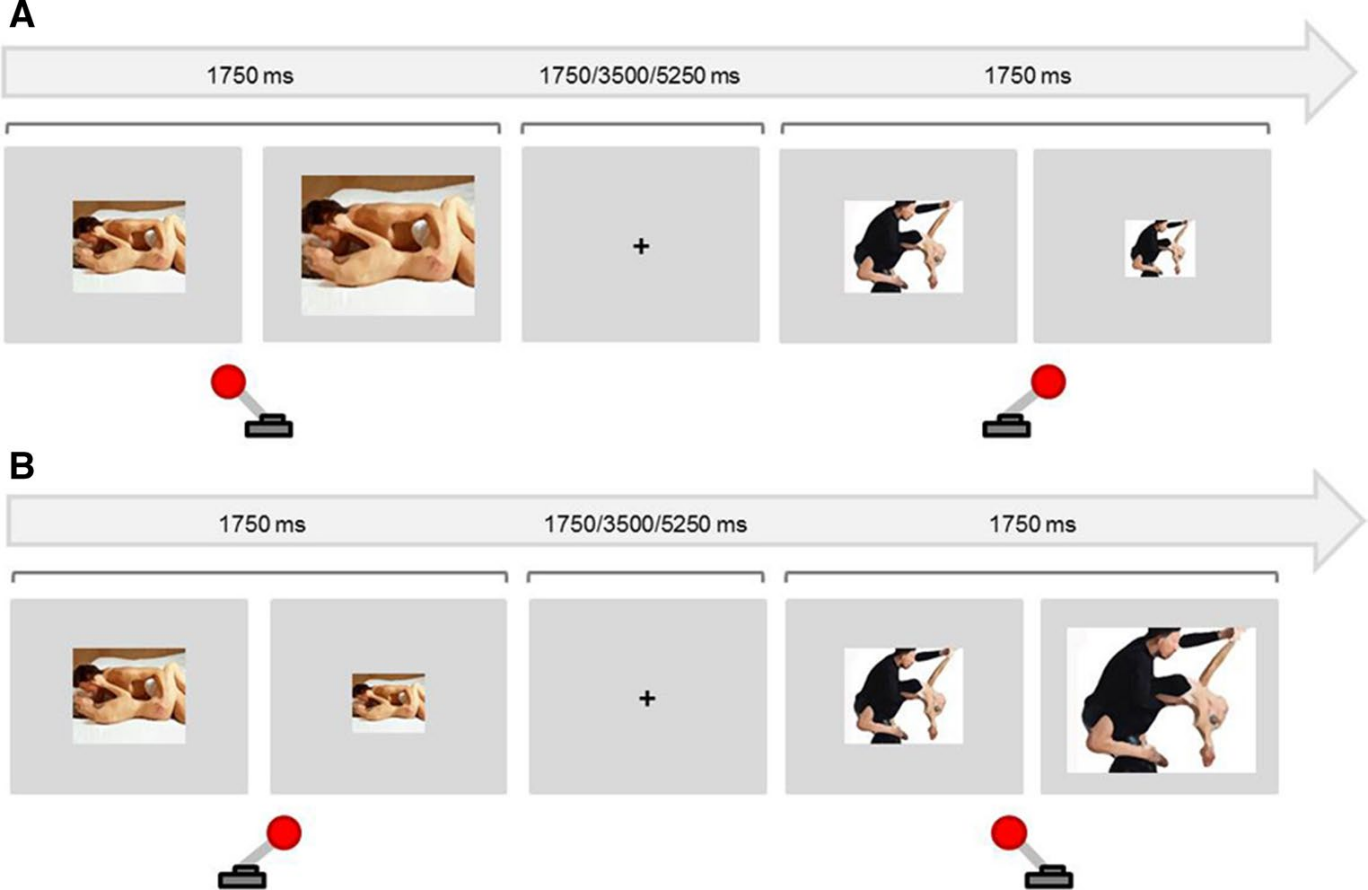
Approach–Avoidance task (AAT). **a** Sex Approach block: Participants were instructed to approach (pull joystick toward themselves) images with sexual content and to avoid (pull joystick away from themselves) non-sexual photographs. Approach caused an increase, while avoidance caused a decrease in image size. **b** Sex avoid block: Participants were instructed to avoid images with sexual content and approach images with non-sexual content. The stimuli shown are dummy photographs only used for illustrative purposes

Sexual stimuli consisted of 48 color photographs displaying sexual intercourse or oral sex between one man and one woman; these pictures were different from those used in the NAP task, and 95% of them were drawn from a validated data set (Rupp & Wallen, 2007). The rest of photographs were selected from the internet on the same basis that the Negative Affective Prime task photographs. The purpose was that every image would be presented only once for every condition (approach–avoid). Non-sexual stimuli consisted of 48 color photographs of one man and one woman dancing (Dewitte, 2016). The proportion of the exposition of the bodies (with special attention to the female body) with respect to the whole picture was comparable in both conditions. In half of the blocks, the photographs were horizontally (337 × 272 pixels) orientated, while in the other half vertically (257 × 400 pixels); this distribution was because there were not enough number of rated photographs with the same orientation. The image resizing halved or doubled the default size. Images were displayed on a light gray background. In two of the four blocks, participants were instructed to approach the sexual stimuli (presented in 50% of the trials included in the block) and to avoid the non-sexual stimuli. For the remaining blocks, they were instructed to do the opposite. Every trial consisted of the presentation of one sexual or non-sexual stimulus for 1750 ms followed by a randomized interval of 1750, 3500, or 5250 ms (Fig. 2). The order of the blocks was counterbalanced. We calculated a Sex Approach–Avoid index, by subtracting the reaction times in Sex Approach blocks from reaction times in Sex Avoid blocks. To control for non-sexual approach–avoidance reaction times, we subtracted the analogous Neutral Approach–Avoidance index, which resulted in the main index used for this task. A larger index indicated a stronger control over sexual motivation, as this implied taking less time to avoid sexual stimuli and/or taking longer to approach them.

#### Go/No-go Task

**Fig. 3:**
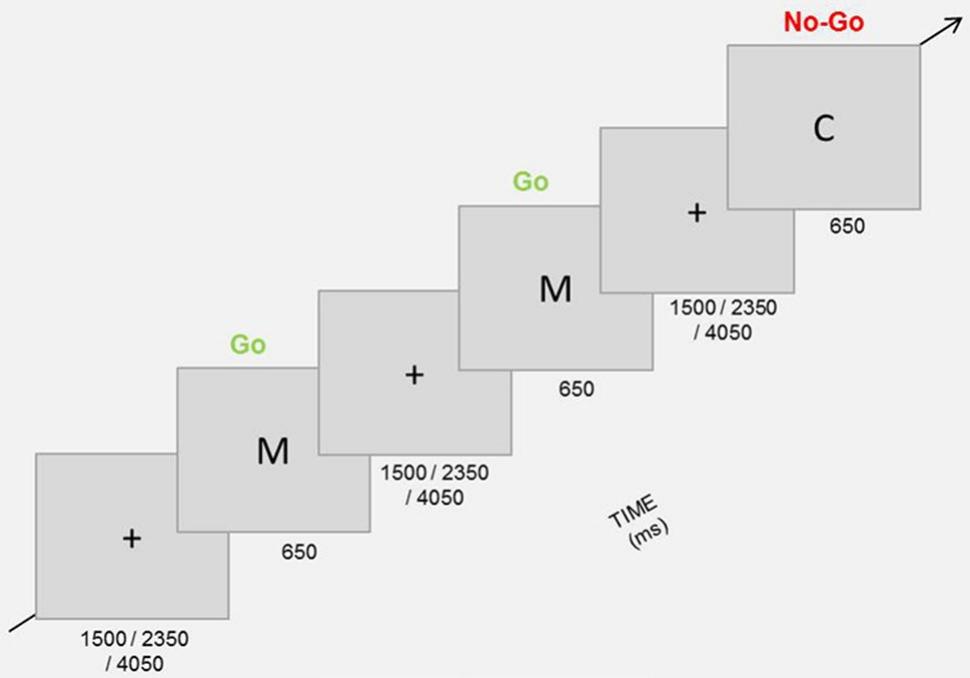
Go/No-go task design

In this task, participants were instructed to press the spacebar when they saw a frequent Go stimulus and to not respond to the infrequent No-go stimulus. As stimuli, the letters “C” and “M” were used and which letter was defined as the Go or No-go stimulus was counterbalanced among participants. White letters (3 × 2.3 cm) were displayed on gray background (adapted from Dambacher et al., 2014). Participants had to complete four blocks of 80 trials each (25% No-go trials). Every trial consisted of the presentation of the stimulus for 650 ms, followed by an inter-trial interval of 1500, 2350, or 4050 ms (Fig. 3). Responses after 650 ms were not registered. Hits (responding to a Go trial), correct No-go’s (not responding to a No-go trial), false alarms (responding to a No-go trial), and misses (not responding to a Go trial) were recorded. A higher number of false alarms indicated a low-response inhibition. The three computer-based tasks were programmed and presented with PsychoPy (Peirce, 2007).

#### Brief Self-Control Scale

This scale consists of 10 items assessing the extent to which an individual is able to regulate his/her own behavior by resisting or inhibiting a preponderant response or desire in order to achieve long-term goals (Example item: “I am good at resisting temptation”). Participants could choose from a 1 to 5-point Likert scale ranging from *Not at all* to *Very much.* BSCS scores range from 13 to 65, with a higher number indicating more self-control. High internal consistency and test–retest reliability have been demonstrated for this scale (Tangney et al., 2004).

#### Sexual Inhibition/Sexual Excitation Scales

This 45-item scale measures the propensity for sexual inhibition and excitation. It contains one factor quantifying sexual excitation (Example item: “When an attractive person flirts with me, I easily become sexually aroused”) and two factors quantifying sexual inhibition: (1) SIS1–Inhibition derived from threat of sexual performance failure, distraction or lack of physical stimulation (Example item: “Once I have an erection, I want to start intercourse right away before I lose my erection”) and (2) SIS2–Inhibition due to the threat of performance consequences, such as risk of being caught or sexually transmitted diseases (Example item: “If I can be seen by others while having sex, I am unlikely to stay sexually aroused”). Response options were on a 4-point Likert scale (ranging from 1 = strongly agree to 4 = strongly disagree; Janssen et al., 2002). The raw scores were inversed in a way that a higher score would indicate a higher excitability (SES, possible range: 20–80) or inhibition (SIS1, possible range: 14–56; SIS2, possible range: 11–44). Previous studies showed solid internal consistency and test–retest reliability for the factors SES, SIS1, and SIS2 (Janssen et al., 2002).

#### Sexual Behavior

In order to have an index of the individual’s sexual cognition and sexual behavior (solitary and dyadic), participants answered according to their experience during the last 4 weeks how often they: (1) thought about sex, (2) watched pornography, (3) masturbated, and (4) had sex (intercourse with penetration). Participants could answer on a 6-point Likert scale, ranging from *Not once* to *Several times a day.*

## Results

Mean and SD for the self-reported measures and sexual behaviors are given in Table 1. Table 2 shows the percentage of participants reporting a determined frequency of sexual thoughts and sexual behaviors during the last 4 weeks. As relationship status was expected to influence different aspects of sexual behavior (e.g., intercourse frequency), the descriptive statistics are shown for the total sample and for partnered and single men separately.

**Table 1:** Descriptive and comparative statistics of partnered and single men

	Total *n* = 52 M (SD)	Partnered *n* = 24 M (SD)	Single men *n* = 28 M (SD)	*t*^p^
Age	24.46 (4.32)	25.08 (4.46)	23.93 (4.21)	ns
Sexual Excitation Scale^a^	50.62 (7.19)	50.58 (6.37)	50.64 (7.9)	ns
Sexual Inhibition Scale 2^b^	29.98 (4.41)	29 (4.14)	30.82 (4.53)	ns
Brief Self-Control Scale^c^	41.46 (7.79)	40.58 (9.23)	42.21 (6.39)	ns
Sexual thoughts	5.06 (.99)	5.29 (.75)	4.86 (1.14)	ns
Masturbation	3.77 (1.06)	3.75 (1.22)	3.79 (.91)	ns
Pornography watching	3.13 (1.21)	2.96 (1.23)	3.29 (1.18)	ns
Intercourse	2.52 (1.46)	3.45 (.21)	1.71 (.24)	− 5.31*

*p < .001

^a^Range: 30–64, ^b^range: 22–43, ^c^range: 27–61

**Table 2:** Percentage of participants reporting a determined frequency of sexual thoughts and sexual behaviors during the last 4 weeks

	Sexual thoughts	Masturbation	Pornography watching	Intercourse
Several times a day	40.4	3.8	0	0
Once a day	32.7	15.4	9.6	7.7
A few times a week	23.1	51.9	40.4	26.9
Once a week	0	13.5	13.5	17.3
One or two times per month	3.8	13.5	26.9	5.8
Not once	0	1.9	9.6	42.3
Total	100	100	100	100

### Computer-Based Tasks

#### Negative Affective Priming Task

**Fig. 4:**
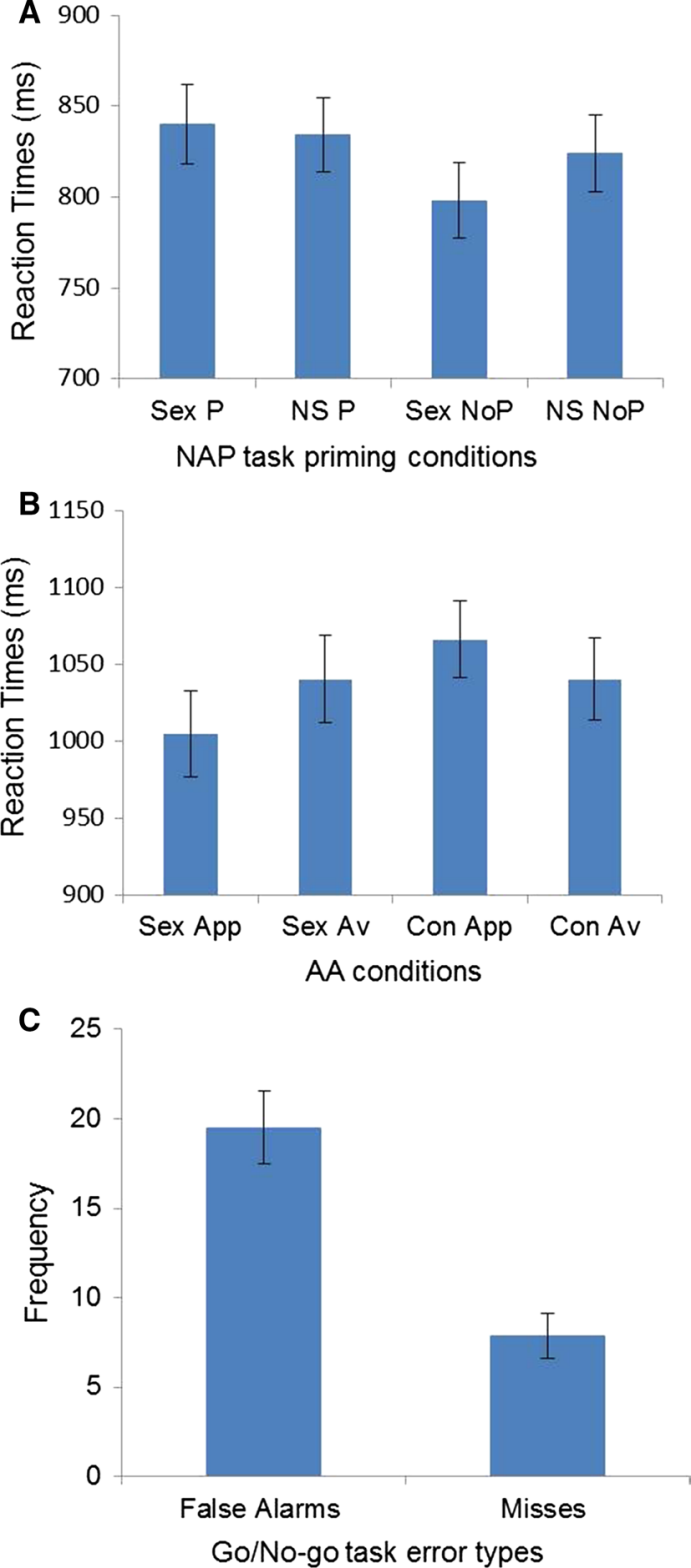
Descriptive statistics of the cognitive tasks. **a** NAP—Negative Affective Priming: Sex P—Sex Priming trials; NS P—Non-Sex Priming trials; Sex NoP—Sex No Priming trials; NS NoP—Non-Sex No Priming trials. **b** AA—Approach–Avoidance task: Sex App—Sex Approach trials; Sex Av—Sex Avoid trials; Con App—Control Approach trials; Con Av—Control Avoid trials

A 2 (Stimuli: Sex vs. Non-Sex) × 2 (Condition: Priming vs. No Priming) ANOVA revealed a main effect of priming condition, *F*(1, 50) = 15.52, *p* = .001; Priming: *M* = 837, SD = 143, No Priming: *M* = 811, SD = 145, and an interaction between stimuli and condition, *F*(1, 50) = 8.15, *p* = .006; Table 3. No main effect was found for stimulus type, *F*(1, 50) = 1.11, *p* = .29; Sex: *M* = 820, SD = 147 ms; Non-Sex stimuli: *M* = 829, SD = 145 (Fig. 4a). Concordant with previous evidence (Dewitte, 2016), the priming effect was significantly larger for sexual than for non-sexual stimuli (*t* = 2.85, *p* = .006; Sexual Priming effect: *M* = 42 ms, SD = 68, Non-Sexual Priming effect: *M* = 10 ms, SD = 53), showing that the omission of visual sexual stimuli requires stronger inhibition.

**Table 3:** Reaction times of the different conditions in the Negative Affective Priming and in the Approach–Avoidance tasks

Negative Affective Priming task M (SD)			
*Sexual*	*Priming*	*Sexual priming*	*Sexual no priming*
820 (147)	837 (143)	840 (155)	798 (147)
*Non*-*sexual*	*No priming*	*Non*-*sexual priming*	*Non*-*sexual no priming*
829 (145)	811 (145)	834 (145)	824 (151)
Approach–Avoidance task M (SD)			
*Sexual*	*Approach*	*Sexual approach*	*Sexual avoid*
1024 (198)	1037 (188)	1004 (202)	1040 (206)
*Non-sexual*	*Avoid*	*Non*-*sexual approach*	*Non*-*sexual avoid*
1055 (183)	1041 (195)	1066 (181)	1040 (194)

#### Approach–Avoidance Task

A 2 (Stimuli: Sex vs. Control) × 2 (Condition: Approach vs. Avoidance) ANOVA revealed that participants were faster responding to sexual than to control stimuli, *F*(1, 50) = 13.93, *p* = .001; Sex trials: *M* = 1024, SD = 198 ms, Control trials: *M* = 1055, SD = 183 ms, which is in concordance with previous evidence (Dewitte, 2016; Hofmann, Friese, & Gschwendner, 2009). Reaction times on approach and avoid trials did not differ, *F*(1, 50) = .28, *p* = .59; Approach trials: *M* = 1037, SD = 188 ms, Avoid trials: *M* = 1041, SD = 195 ms, and there was a significant interaction between stimuli and condition, *F*(1, 50) = 8.07, *p* = .006, indicating a faster response approaching sexual stimuli over the other conditions (Table 3; Fig. 4b).

#### Go/No-go Task

Participants committed on average 19.51 (SD = 14.62) false alarms and 7.86 (SD = 8.91) misses (Fig. 4c). On false alarms, reaction times were faster than on Go trials (False alarms: *M* = 242, SD = 36 ms, Go trials: *M* = 358, SD = 34 ms; *t* = − 15.49, *p* = .001). For further analyses, false alarms were log-transformed to correct skewness. To examine whether relationship status would influence performance on the implicit measures, we conducted a series of tests, which revealed that single and partnered men did not differ significantly in their performance on none of the three tasks.

### Sexual Inhibition and General Inhibition

None of the relationships between general inhibition (Go/No-go task), self-control (BSCS), sexual cognitive inhibition (NAP), sexual motor inhibition (AA), and sexual inhibition as a trait (SIS2) were significant (Table 4).

**Table 4:** Correlational statistics between sexual inhibition, general inhibition, and self-control

	Sexual inhibition			General inhibition and self-control	
	NAP CI (*p*)	AA CI (*p*)	SIS2 CI (*p*)	GNG FA log CI (*p*)	BSCS CI (*p*)
NAP	1	.07 [− .12, .32] (.59)	.01 [− .23, .29] (.97)	.03 [− .28, .35] (.86)	− .21 [.45, .07] (.13)
AA		1	.6 [− .21, .32] (.68)	− .01 [− .26, .18] (.91)	.04 [.17, .25] (.77)
SIS2			1	.15 [− .12, .43] (.28)	.04 [.17, .25] (.77)
GNG FA log				1	− .17 [.45, .04] (.21)
BSCS					1

*NAP* Negative Affective Priming task, *AA* Approach–Avoidance task, *SIS2* Sexual Inhibition Scale factor 2, *GNG FA log* Go/No-go false alarms (log-transformed), *BSCS* Brief Self-Control Scale. *CI* confidence intervals (95%, bootstrap resamples = 1000). In brackets: lower and upper limits. (*p*) Alpha value

### Sexual Motor Inhibition, Sexual Cognitive Inhibition, and Sexual Inhibition as Trait

To investigate whether sexual motor inhibition, sexual cognitive inhibition, and sexual inhibition as a trait were related, we performed correlation analyses (Table 4). There were no significant relationships among these processes and trait.

### Regulatory Mechanisms of Sexual Thoughts and Sexual Behavior

To investigate whether sexual inhibition, general inhibition, self-control, and sexual excitation were related to the frequency of different sexual manifestations, we performed correlation and multiple regression analyses. In Table 5, the correlation among these variables derived from the computer-based tasks and the self-reports is shown. We conducted separated analyses to predict the frequency of sexual thoughts, solitary sexual behavior (pornography watching and masturbation), and dyadic sexual behavior (intercourse).

**Table 5:** Correlational statistics between sexual inhibition, general inhibition, and sexual excitation measurements, and sexual manifestations frequency

	Tasks			Self-reports		
	NAP CI (*p*)	AA CI (*p*)	GNG FA log CI (*p*)	SES CI (*p*)	SIS2 CI (*p*)	BSCS CI (*p*)
Sexual Thoughts	− .36 [− .57, − .13] (.01)	.13 [− .21, .47] (.32)	.02 [− .26, .35] (.84)	.26 [.001, .48] (.06)	− .01 [− .28, .25] (.92)	− .04 [− .32, .21] (.77)
Masturbation	− .14 [− .38, .08] (.32)	.15 [− .21, .41] (.27)	.04 [− .23, .37] (.75)	− .004 [− .23, .24] (.97)	− .11 [− .39, .21] (.47)	− .23 [− .48, .04] (.08)
Pornography	− .04 [− .29, .21] (.76)	.27 [− .05, .52] (.04)	− .03 [− .34, .29] (.83)	.06 [− .18, .35] (.62)	− .16 [− .36, .11] (.25)	− .26 [− .51, .01] (.06)
Intercourse	.01 [− .23, .32] (.91)	.08 [− .36, .21] (.56)	.23 [− .04, .49] (.11)	.11 [− .11, .38] (.46)	− .31 [.05, .52] (.02)	.03 [− 28, .21] (.83)

*NAP* Negative Affective Priming Task, *AA* Approach–Avoidance task, *GNG FA log* Go/No-go false alarms (log-transformed), *SES* Sexual Excitation Scale, *SIS2* Sexual Inhibition Scale factor 2, *BSCS* Brief Self-Control Scale. *CI* confidence intervals (95%, bootstrap resamples = 1000). In brackets: lower and upper limit. (*p*) Alpha value

To predict the frequency of sexual thoughts, we entered the Sexual Cognitive Inhibition (NAP) index and Sexual Excitation (SES) scores to the model. To predict the frequency of pornography watching, masturbation, and intercourse, we entered the sexual motor inhibition (AA) and general inhibition (Go/No-go–log-transformed number of false alarms) indices, along with the self-control (BSCS), sexual inhibition (SIS2), and sexual excitation (SES) trait scores in separate models. To exclude the fact that sexual cognitive inhibition (NAP) would predict the frequency of sexual behavior or that sexual motor inhibition (AA) and sexual inhibition as a trait (SIS2) would predict the frequency of sexual thoughts, we repeated the same analyses including both sexual inhibition processes and sexual inhibition as trait into the models. These variables were included stepwise into the model as we did not have a priori predictions about which variables would be more relevant in predicting the different sexual manifestations.

**Fig. 5:**
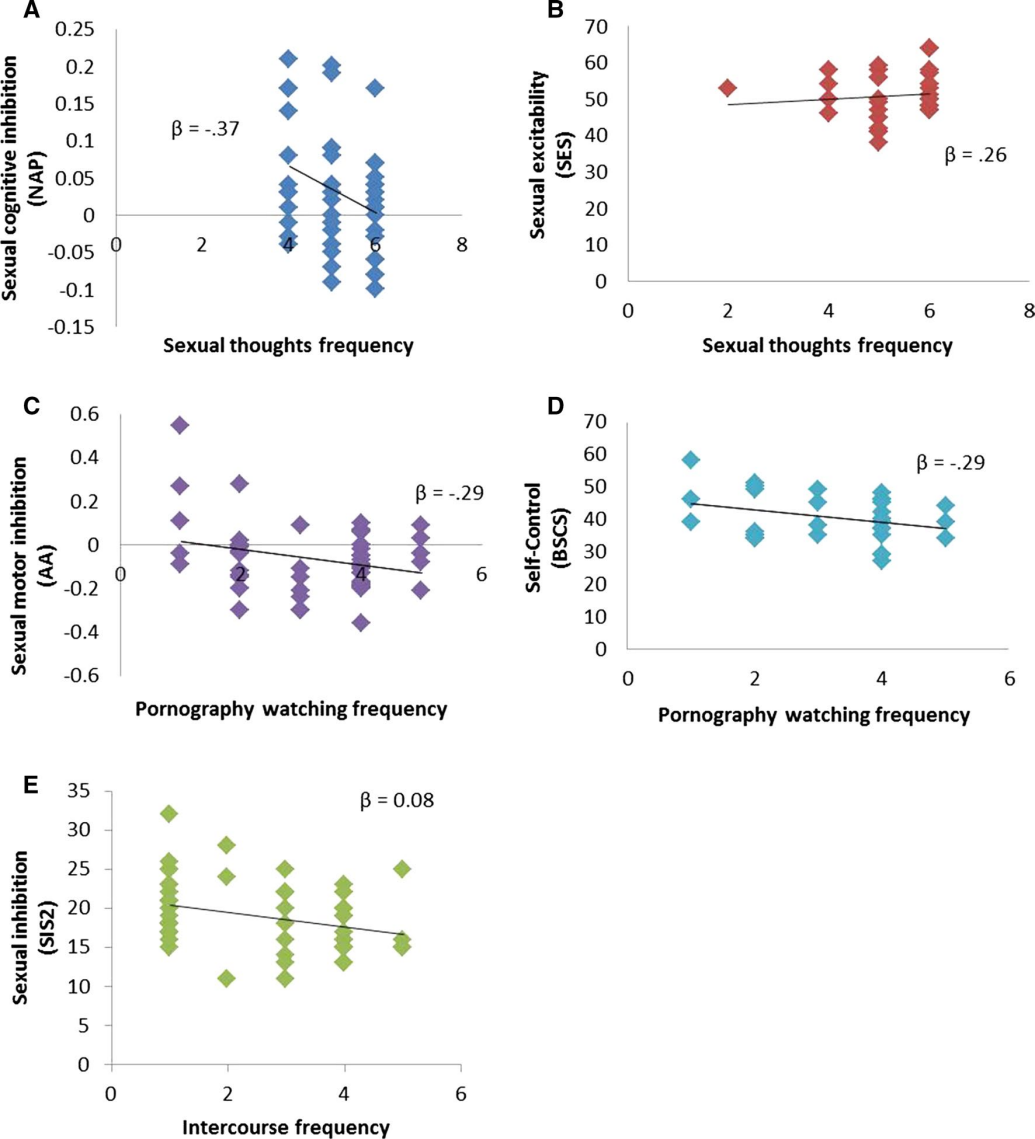
Significant predictors of the frequency of sexual thoughts, individual sexual behavior, and intercourse

The regression analysis showed that NAP and sexual excitability predicted the frequency of sexual thoughts (*R*^2^ = .21; *p* = .005; Table 6; Fig. 5a, b). Results indicated that a higher frequency of sexual thoughts was associated with a lower sexual priming effect (less sexual cognitive inhibition) and a higher sexual excitability. These results did not change when sexual motor inhibition (AA) and sexual inhibition as trait (SIS2) were entered into the model. None of the variables significantly predicted the frequency of masturbation. The frequency of watching pornography, on the other hand, was inversely predicted only by sexual motor inhibition (AA) and the degree of self-control (BSCS) (*R*^2^ = .16; *p* = .016; Table 6; Fig. 5c, d). The self-reported sexual inhibition due to sexual performance consequences (SIS2) inversely predicted the frequency of intercourse (*R*^2^ = .08; *p* = .04; Table 6; Fig. 5e). Including sexual cognitive inhibition (NAP) in the models to predict sexual behavior (masturbation, pornography watching, and intercourse frequency) did not add a predictive value.

**Table 6:** Multiple regression models predicting sexual thoughts, individual sexual behavior, and intercourse

	*β*	*T*	*p*
*Sexual thoughts*			
Negative Affective Priming task Sexual Excitation Scale	− .37 .26	− 2.8 2.01	.007 .05
*Masturbation*			
	–	–	ns
*Pornography watching*			
Approach–Avoidance task Brief Self-Control Scale	− .29 − .29	− 2.2 − 2.16	.03 .03
*Intercourse*			
Sexual Inhibition Scale 2	− .29	− 2.1	.04

## Discussion

In the current study, we aimed to deepen our understanding of the inhibitory mechanisms that are potentially involved in regulating sexual cognition and behavior. First, we aimed to study whether sexual inhibition was related to general inhibition, measured both as processes and as traits. Additionally, we examined whether sexual inhibition would comprise distinct mechanisms that would work at different levels. Here, we considered cognitive and motor-motivational processes and whether these would relate to sexual inhibition as a trait. Finally, we examined whether the different inhibitory processes and traits (general and sexual) along with sexual excitation as a trait distinctively predicted the frequency of two spheres of sexuality: thoughts and behavior.

### Sexual Inhibition and General Inhibition

Regarding the question of whether sexual inhibition and general inhibition as traits and processes would constitute different or related constructs, our results showed no significant relationship between sexual and general inhibition as processes. Contrary to our prediction in Hypothesis 1, the ability to inhibit a sexually driven response (AA) seems independent from the general ability to suppress a preponderant response (Go/No-go). Similarly, we did not find support for Hypothesis 2 which predicted that the general ability to control behavior regarded as trait (self-control) would relate to the degree to which individuals inhibit their sexual arousal due to the risk of undesired consequences (SIS2). Altogether, these findings do not support that sexual inhibition derives from a general inhibitory domain and instead requires specific cognitive mechanisms. Although general inhibition deficits can impact on sexual behavior as seen in frontal brain damage patients (Baird et al., 2007), our results indicate that additional mechanisms exist to inhibit different sexual manifestations.

### Sexual Motor Inhibition, Sexual Cognitive Inhibition, and Sexual Inhibition as Trait

Hypothesis 3 predicted that sexual cognitive inhibition and sexual motor inhibition were not related, which was supported by our results. The sexual priming effect (measuring sexual cognitive inhibition) involves the capacity to inhibit sexual visual information. With this regard, it had been debated whether the negative priming effect reflected retrieval or inhibition processes (Frings, Schneider, & Fox, 2015; Mayr & Buchner, 2007). However, inhibition and retrieval are intrinsically linked in the negative priming effect: Retrieving the content type of the distractor from the prime trial is a prerequisite for the inhibitory process to happen in the probe trial. In addition, we controlled for a neutral negative priming effect, in which the retrieval process was equivalent, and therefore, we can imply that we specifically targeted the inhibitory component.

In regard to sexual motor inhibition, the active avoidance of sexual stimuli implies control over the motor system to inhibit an inner reward-driven response. Typically, the automatic appraisal of a rewarding stimulus evokes approach tendencies. Avoiding this stimulus requires control over those motivational motor tendencies. This inhibitory component is reflected by the delayed response times in avoiding sexual stimuli as compared to approaching them. Thus, sexual cognitive inhibition and sexual motor inhibition comprise different processes and the lack of relationship between them supports the idea that there are different mechanisms to regulate sexual cognition and behavior. This diversity may constitute the core of the complexity and variety of sexual disorders.

Contrary to what was predicted in Hypothesis 4, the ability to inhibit sexually driven motor responses (AA) did not relate to sexual inhibition as a trait (SIS2). This may indicate that the Approach–Avoidance paradigm is not sensitive enough to reflect the inhibition of sexual arousal in quotidian circumstances. It is also possible that what is measured by this factor of the SIS (loss of sexual arousal and erection due to fear of sexual performance consequences) is inherently different from the motor response to approach or avoid a sexual stimulus; whereas the latter constitutes an intentional action, the former one refers to the automatic body response as a product of fear of the sexual performance consequences (even if the fear derives from a realistic risk).

### Regulatory Mechanisms of Sexual Thoughts and Sexual Behavior

Finally, we examined whether the different inhibitory processes and traits, on the one hand, and sexual excitability, on the other hand, would distinctively predict specific sexual expressions. We expected that the sexual cognitive inhibition would predict the frequency of sexual thoughts (Hypothesis 5). Our results supported this hypothesis, which is in line with the process model of emotion regulation (Gross, 2002), stating that successful attentional direction prevents the emotional response (arousal) to take place, thereby preventing the sexual thoughts to interfere with quotidian goals. The fact that the sexual priming effect predicted only sexual thoughts but not sexual behavior, points to the specificity of this cognitive process and suggests that inhibiting sexual input information at a cognitive level may not be central in inhibiting actual overt behavior.

To inhibit sexual behavior, other inhibitory mechanisms are needed. Hypothesis 6 predicted that sexual motor inhibition and general inhibition as processes would directly relate to sexual behavior. The ability to inhibit sexually driven motor responses (sexual motor inhibition) predicted the frequency of watching pornography. This finding is in line with previous research showing that this task correlates with sexual behavior at the implicit level (viewing time of erotic stimuli) but not at the explicit level (choosing a sex calendar over an art calendar) (Hofmann et al., 2009). It seems that the sexual AA reveals an inner motivation that is not overtly expressed but is still manifested at the individual behavior level.

General motor response inhibition (Go/No-go false alarms) did not predict the frequency of any sexual manifestation. This was unexpected, as in a previous study sexually compulsive individuals committed more false alarms and misses in the same task (Miner et al., 2009). It is possible that the relationship between general motor response inhibition and sexual inhibition is only present in extreme cases and that our sample did not include enough of these cases (individuals with a high frequency of sexual manifestations) to show this relationship. In this way, high or medium general inhibition may not be necessarily associated with high or medium sexual inhibition, but only extremely low general inhibition may be related to extremely low sexual inhibition. Furthermore, a previous study showed that in addition to self-reported impulsivity, the level of abstract intelligence was associated with the commission of false alarms in a Go/No-go task under a sexual arousing condition (Macapagal et al., 2011). Thus, it may not be the failure in general inhibition alone but its combination with other factors which causes a failure in sexual inhibition.

Hypothesis 7 predicted that inhibitory traits (sexual inhibition and self-control) would also predict sexual behavior. Self-control was also a significant predictor of the frequency of watching pornography. In quotidian life, one should overcome pleasant temptations in order to achieve mid- and long-term goals. In this regard, self-control is relevant to regulate a behavior that can be highly rewarding, and unlike intercourse, risk-free and easily accessible.

The frequency of intercourse was predicted by sexual inhibition as a trait (SIS2). This construct emphasizes the decrease in sexual arousal and erectile response under the risk of future consequences. Therefore, the inhibition of sexual physiological responses occurring through higher-order processing (anticipation of negative consequences or moral cognition) seems crucial in the regulation of dyadic sex. This is relevant since the failure of this inhibitory mechanism would presumably lead to harmful consequences for the individual and for others. In fact, this factor has been previously related to risky sexual behavior (Bancroft et al., 2004; Janssen & Bancroft, 2007). Additionally, this factor (SIS2) did not predict non-dyadic sexual aspects (i.e., sexual thoughts, masturbation, or pornography watching), which is not surprising as this trait taps into the inhibition involved when there is a threat, and non-dyadic sexual behaviors rarely pose any evident risk or danger. Noticeably, no process or trait was able to predict the frequency of masturbation. It is possible that this individual sexual behavior is more influenced by mood regulation and sexual attitudes (Bancroft & Vukadinovic, 2004).

Finally, Hypothesis 8 predicted that sexual excitability would be a significant predictor of sexual thoughts and sexual behavior. Remarkably, sexual excitability predicted only the frequency of sexual thoughts. Because sexual thoughts are not manifest and belong to one’s inner fantasy world, they require less inhibitory efforts to accomplish social and moral requirements and, thus, might be more susceptible to arousability levels. The fact that sexual excitability was not a predictor of either individual or dyadic sexual behavior emphasizes the dominant role of inhibitory and regulatory mechanisms in the expression of sexual behaviors. In this sense, while excitability and sexual inhibition modulate the amount of sexual thoughts, the behavior itself seems rather regulated by inhibitory mechanisms, which would imply that individuals who are easily aroused do not necessarily engage in more sexual behavior. Instead, the sexual behavior of individuals would depend mainly on inhibition and self-control rather than excitability levels, with different inhibitory processes regulating different aspects of sexual thoughts, desire, and behavior.

### Limitations

This study supports the idea that multiple inhibitory mechanisms exist in the regulation of sexual cognition and behavior. These different inhibitory mechanisms modulate distinct aspects of sexual behavior and cognition and are at play during different phases of sexual regulation. Nonetheless, some aspects limit the generalization of our results. The sample of this study was mainly composed of Caucasian university students. It is, however, noteworthy that the study background of our participants was rather heterogeneous (e.g., medicine, engineering, business, psychology, economy). For ethical reasons, the participants were aware that the study would include erotic material which could also have resulted in a biased self-selected sample.

In addition, as occurs with small samples, the current study entails the risk of false negative results. However, the alpha values of the non-significant results were substantially far beyond the threshold of significance. Another problem of small samples is the potential inaccuracy in the magnitude of the correlation values (Schönbrodt & Perugini, 2013). In this regard, the confidence intervals generated by the bootstrapping procedure (Tables 3 and 4) indicated that the significant correlation estimates were for the most part within the same direction (indicating either positive or negative relationships). This way, we cannot reassure the accuracy of the correlation parameters magnitude but we can have an approximation of its variability.

Although the present study shows that computer-based tasks can advance our understanding of the regulation of sexual behavior, we are also aware that these methods along with self-reports can capture only partial components of sexual cognition. Regarding sexual arousability, for example, we based our conclusions on merely one explicit measure (Sexual Excitability Scale). Future studies can include additional physiological indicators of sexual arousability (e.g., heart rate, skin galvanic response, or penile plethysmography) to have a more integrative perspective.

Although we tried to include ecologically valid measurements for sexual behavior (frequency of sexual thoughts, masturbation, pornography watching, and intercourse), we assessed those only via self-reports. To study sexual inhibition and sexual behavior, future studies can also consider to include behavioral non-computer-based measurements (for examples, see Gailliot & Baumeister, 2007; Hofmann et al., 2009) in order to achieve higher ecological validity and reliability. Finally, we studied sexual regulatory mechanisms in healthy participants and regarding healthy sexual behaviors. It remains to be investigated whether the same mechanisms are involved in undesired or unhealthy sexual behavior.

### Conclusion

This work aimed to study the relationship between different inhibitory mechanisms and their specific role in regulating sexuality at the cognitive and at the behavioral level. The presented evidence supports the notion that sexual inhibition is distinct from one’s general ability of inhibitory control. Along with clinical observations in brain damage patients showing that sexual disinhibition is not always accompanied by a general inhibition deficit (Baird et al., 2007), these results support the existence of different inhibitory mechanisms in the regulation of sexual cognition and behavior. Furthermore, in this study, we observed that sexual cognitive inhibition is not related to sexual motor inhibition and that both processes distinctively predicted different facets of sexuality (sexual thoughts and sexual behavior). Following the process model of emotion regulation (Gross, 2002), we propose that whereas sexual cognitive inhibition prevents the sexual arousal to take place, the sexual motor inhibition is involved to control an ongoing sexual emotional reaction. Future studies may examine whether the distinctive involvement in the examined mechanisms underlies specific symptomatology and comorbidity in sexual disorders.
